# The environment ontology: contextualising biological and biomedical entities

**DOI:** 10.1186/2041-1480-4-43

**Published:** 2013-12-11

**Authors:** Pier Luigi Buttigieg, Norman Morrison, Barry Smith, Christopher J Mungall, Suzanna E Lewis

**Affiliations:** 1 HGF-MPG Research Group on Deep-Sea Ecology and Technology, Alfred Wegener Institute, Helmholtz Centre for Polar and Marine Research, Am Handelshafen 12, Bremerhaven 27570, Germany; 2 Genomics Division, Lawrence Berkeley National Laboratory, Berkeley, CA 94720, USA; 3 Department of Philosophy, University at Buffalo, Buffalo, NY 14260-4150, USA; 4 School of Computer Science, The University of Manchester, Oxford Road, Manchester M13 9PL, UK

**Keywords:** Environment, Ecosystem, Biome, Ontology

## Abstract

As biological and biomedical research increasingly reference the environmental context of the biological entities under study, the need for formalisation and standardisation of environment descriptors is growing. The Environment Ontology (ENVO; http://www.environmentontology.org) is a community-led, open project which seeks to provide an ontology for specifying a wide range of environments relevant to multiple life science disciplines and, through an open participation model, to accommodate the terminological requirements of all those needing to annotate data using ontology classes. This paper summarises ENVO’s motivation, content, structure, adoption, and governance approach. The ontology is available from http://purl.obolibrary.org/obo/envo.owl - an OBO format version is also available by switching the file suffix to “obo”.

## Background

Biologically motivated research is generating [1-3] and archiving [4,5] ever-larger quantities of computerised data from environmental samples. Simultaneously, biomedical researchers have begun to take particular interest in the physical environment of organisms at all scales, from microbes to patients [6-9], while scientists in epidemiology and public health are developing a stronger interest in location- and environment-based information for purposes of disease tracking [10,11]. In these complex and data-rich fields, the need to describe systematically the environmental context of biological entities is being increasingly acknowledged as a means to mobilise data for environment-aware analyses (see e.g. [12]).

It was the need for consistent description of the environmental origins of tissue, pathogen, and metagenomics samples, together with a parallel need in the labeling of samples and artifacts in museum collections that precipitated the creation of the Environment Ontology (ENVO). A series of meetings and workshops laid the foundation for addressing these needs by establishing the ENVO consortium and the ontology itself. ENVO is comprised of classes (terms) referring to key environment-types that may be used to facilitate the retrieval and integration of a broad range of biological data. In developing ENVO, we recognized the many existing resources which address, among other entities, environment-types [13-16] and were motivated by the value of unifying such resources in a foundational, or building block, ontology developed within a federated framework and exclusively concerned with the specification of environment types, independent of any particular application. Thus, ENVO was developed with the goal of interoperability with the numerous biological and biomedical ontologies compliant with Open Biomedical and Biological Ontologies (OBO) Foundry principles [17,18] and is being aligned to the Basic Formal Ontology (BFO 2.0 [19]; see below) in aid of semantic homogeneity. Lastly, ENVO is designed as an open project, poised to respond to the needs of its users and draw from their insights. We hope that ENVO will offer benefits similar to those of the Gene Ontology (GO; [20]) in allowing a standardized and semantically controlled representation of a domain central to life science research in an open, community-led manner.

Classes describing natural environments currently dominate ENVO’s content as the ontology is geared towards use in the biological domain. Nevertheless, ENVO is suitable for the annotation of any record that has an environmental component. For example, one may use ENVO classes to provide information on the environment of remote sensing devices or of photographic image content. Indeed, classes corresponding to man-made objects, for example *hypodermic needle* [ENVO_ 02000000]^a^, *umbrella* [ENVO_ 02000052], or *terrarium* [ENVO_00000349], are included in the ontology. Further, ENVO offers terminology resources both for specialists and for non-experts, a feature particularly useful in scenarios where citizen scientists and volunteers are involved in sampling or observational campaigns (for example as described in [21]).

In this paper, we briefly describe ENVO’s current content, structure, adoption, and governance model in order to orient potential users and contributors. Readers should be aware that ENVO is a living ontology shaped by multiple contributors and thus subject to change. However, the ontology is under version control in a Google Code repository [22] and historical changes are fully tracked. More information is present in the Downloads section, below.

## Results and discussion

In what follows, ontology classes (or synonymously, ‘terms’), written in italics, are taken from ENVO unless otherwise marked through the provision of an appropriate namespace, as in ‘PATO:*cellular motility*’. The namespace and unique identifier of each term’s OBO Foundry Uniform Resource Identifier, e.g. ‘ENVO_00002297’ for *environmental feature*, will be included on first mention of any class. Full URIs are of the form: http://purl.obolibrary.org/obo/ENVO_00002297, and are resolved to OWL as well as to human-readable web pages.

### Semantics of environment terms

While all biologists have an intuitive understanding of what is meant by ‘environment’, a rigorous definition of this class is non-trivial (see e.g. [23,24]). For example, when taken simply as the “surrounding space” of an entity, the causal relevance of an environment to that entity as well as its boundaries are unclear. Consider a population of humans in Biosphere 2 [25,26]. While it is surrounded by the Santa Catalina Mountains (AZ, USA), many environmental factors of this region have little relevance to this population’s biology and behaviour. The ecosystems within Biosphere 2, however, are of greater causal relevance and thus more appropriately identified as the population’s environments. Further, confusion often arises when attempting to distinguish an environment from a habitat or niche: the environment an organism was observed in or isolated from may have little to do with its habitat or its niche, as described, for example, in [27].

In an effort to clarify these concepts, work has been done to align ENVO’s four top-level classes to classes from the Basic Formal Ontology (BFO; [19]), an upper-level ontology that provides a semantic foundation for a wide range of domain ontologies^b^. Through this exercise, a new subclass of BFO:*material entity* [BFO_0000040], *system*, has been proposed to describe causally integrated yet multi-component entities such as environments. We propose that an environment (synonymous with an *environmental system* [ENVO_01000254]) is a certain sort of system which has the disposition to environ, that is to contain within its BFO:*site* [BFO_0000029] and causally integrate, some BFO:*material entity*. Examples of environments range from rainforests to gut lumens to the interiors of virally infected cells. As described below, the subclasses of *environmental system* will reference environment-types familiar to most biologists.

ENVO’s *biome* [ENVO_00000428] and *habitat* [ENVO_00002036] classes are subclasses of *environmental system*. The *biome* class represents environmental systems to which resident ecological communities have evolved adaptations. Thus, a *biome* may be thought of as a community-centric ecosystem, whose extent is defined by the presence of the communities adapted to it. This requires that a *biome* possesses a degree of spatial and temporal stability that has allowed at least some of its constituent communities to adapt. Classes such as *tundra biome* [ENVO_01000180] and *coniferous forest biome* [ENVO_01000196] are included in ENVO. Currently, the biome branch of the ontology makes no commitment to a specific spatial or temporal scale. While biomes are community-centric, ENVO treats habitats in a population-centric manner: habitats refer to environmental systems which include those components needed to allow the survival and growth of a specific ecological population. Our objective is to differentiate between habitats and other environment types following considerations such as those in [18]. The subclasses of ENVO’s *habitat* class are currently under review.

The environment-types described above are useful in ecological settings; however, environments are often described by referencing a single entity that has a strong causal influence on its surrounding space. For example, a coral reef environment is determined by the presence and influence of a *coral reef* [ENVO_00000150]. Similarly, the human gut environment is determined by the human gut. Removal of either the *coral reef* or the human gut would cause the associated environmental system to collapse. Environmental systems of this kind make no specific reference to ecological communities or populations (as do biomes and habitats resp.), but to some central, supporting ‘feature’. Entities that act in this way as the causal ‘hubs’ or supports of a given environmental system are referenced by classes in ENVO’s top-level *environmental feature* [ENVO_00002297] hierarchy. For example, the environmental feature *seamount* [ENVO_00000264] would support a seamount environment, i.e. an environmental system which is supported by, and whose properties are determined by, the presence of a seamount. Currently, ENVO only includes classes for environmental features and not the environmental systems associated with them. Work to arrive at a formal definition of *environmental feature* is ongoing. Current considerations are focused on differentiating the *environmental feature* class from the BFO:*material entity* class by defining a BFO:*role* [BFO_0000023] which declares the environment-supporting nature of a *environmental feature.*

In contrast to the classes above, which identify countable entities, the subclasses of the top-level *environmental material* [ENVO_00010483] class refer to masses, volumes, or other portions of some medium included in an environmental system (for a full discussion of ‘medium’ see: [28]). A portion of *environmental material* is understood to be more complex and variable in composition than a simple collection of material entities (e.g. a collection of silicate particles). For example, the environmental material *soil* [ENVO_00001998] typically contains aggregates of fine rock particles, sand grains, clay particles, silt particles, communities of animals, plants, fungi and microbes, small parts of organisms, organic matter, water inclusions, and airspaces. As is the case with environmental feature, work on the definition of this class is ongoing. This class is likely to be defined as a subclass of BFO:*fiat object* [BFO_0000024] which forms the medium or part of the medium an environmental system.

**Figure 1:**
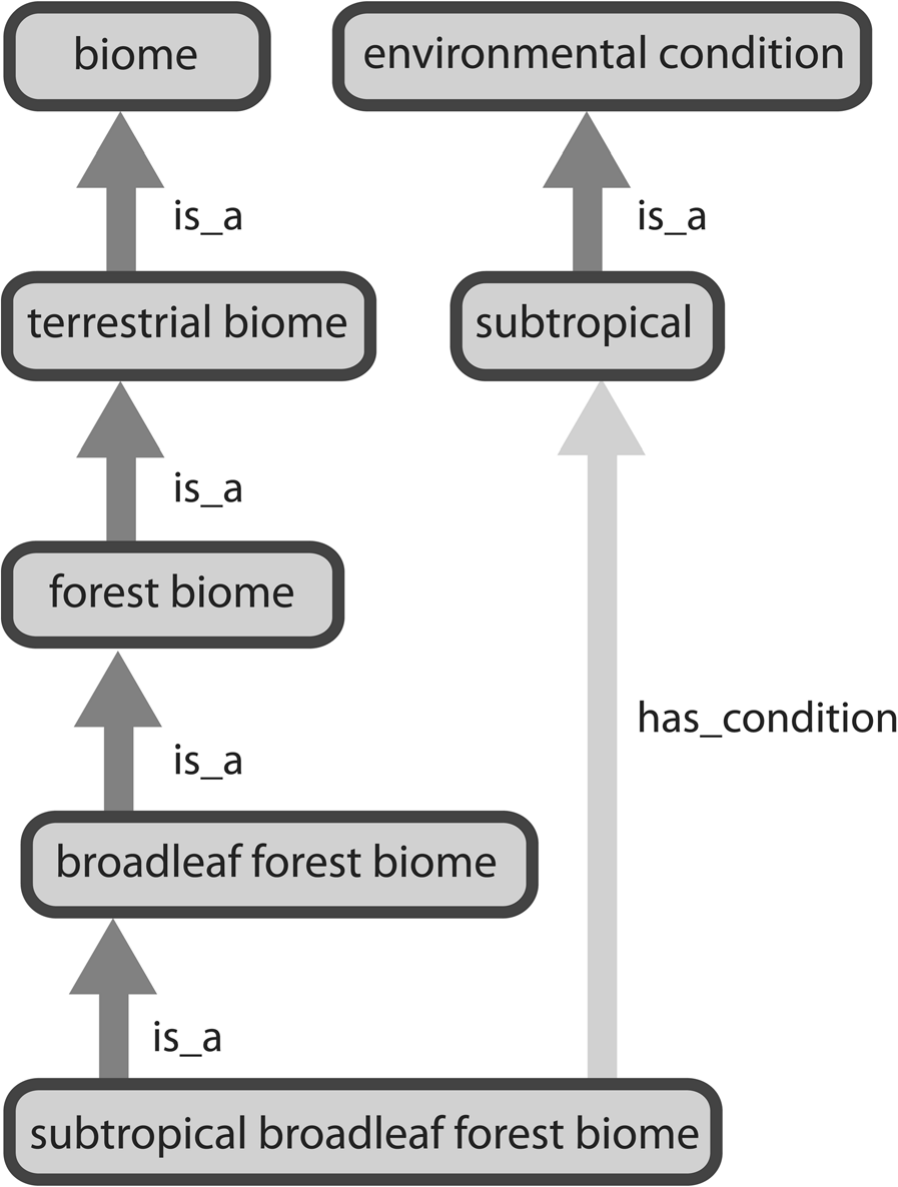
**Subclasses of ENVO’s *environmental condition *may be used as differentiae when defining subclasses of classes in the *biome *(shown)*, environmental feature, or environmental material *hierarchies.** Retrieval of entities annotated with ENVO classes that satisfy a given condition is thus facilitated.

Lastly, ENVO includes the top-level class, *environmental condition* [ENVO_01000203]. Subclasses of *environmental condition* define specific ranges of determinate qualities (e.g. a temperature range of 20 – 37°C, a solar irradiation range of 426 W/m^2^ - 773 W/m^2^) or combination of qualities that are present in an environmental system. These may be used as differentiae with *biome*, *environmental feature*, or *environmental material* classes as genera. For example, the class *subtropical broadleaf forest biome* [ENVO_01000201], includes the differentia has_condition *subtropical* [ENVO_01000205] (Figure 1). Note that subclasses of *environmental condition* such as *tropical, temperate* [ENVO_01000206], and *polar* [ENVO_01000238] are intended to reflect qualities such as the degree of solar irradiation received by an environment rather than reference geographic regions. A complete definition of these classes has yet to be finalised and will be derived from BFO:*quality* [BFO_0000019].

Where possible, the semantics of ENVO classes are established using references to classes in other, related ontologies. For example, the *environmental material* class *xylene contaminated soil* [ENVO_00002146] has a genus-differentia definition with the genus *contaminated soil* [ENVO_00002116] and differentia: has_increased_levels_of CHEBI:*xylene* [CHEBI_27338].

We acknowledge that our treatment of terms such as *biome* and *habitat* may cause debate and we welcome criticism and suggestions for revision. One of ENVO’s central goals is to standardise the often loose usage of such terms across numerous domains, including not only ecology and environmental biology but also multiple other geospatial sciences. The current top-level classes represent an attempt to create such an initial standardization and to present it for community review with the goal of achieving wider consensus. In the interim, measures to map different usages to the appropriate ENVO class by making extensive use of synonyms are being developed.

### Architecture and growth

In this section, ENVO’s *biome*, *environmental feature*, and *environmental material* hierarchies – which are the ontology’s most developed branches and are of primary interest to annotators – are briefly described.

ENVO’s *biome* hierarchy currently recognizes two immediate subclasses: *terrestrial biome* [ENVO_00000446] and *aquatic biome* [ENVO_00002030]. Most subclasses of *terrestrial biome* have been adapted from the list of terrestrial “major habitat types” defined by the World Wide Fund for Nature (WWF; http://worldwildlife.org/biomes/; [29]). However, the *anthropogenic terrestrial biome* [ENVO_01000219] branch of the ontology is being gradually extended with classes adapted from the classification of Ellis et al. [30,31]. The *aquatic biome* class has two subclasses, namely the *marine biome* [ENVO_00000447] and *freshwater biome* [ENVO_00000873] classes. The former hierarchy has been developed in some detail with input from marine scientists and includes classes representing depth-dependent layers of the oceans and seas as well as biomes associated with geographic entities (e.g. *epeiric sea biome* [ENVO_01000045]). The *freshwater biome* branch is in a considerably less developed state and includes subclasses adapted from the WWF’s freshwater ecosystem classification. Classes such as *Small river biome* [ENVO:00000890] and *Large river biome* [ENVO:00000887], which are of ambiguous and relative scale, are in need of curation or replacement.

**Figure 2:**
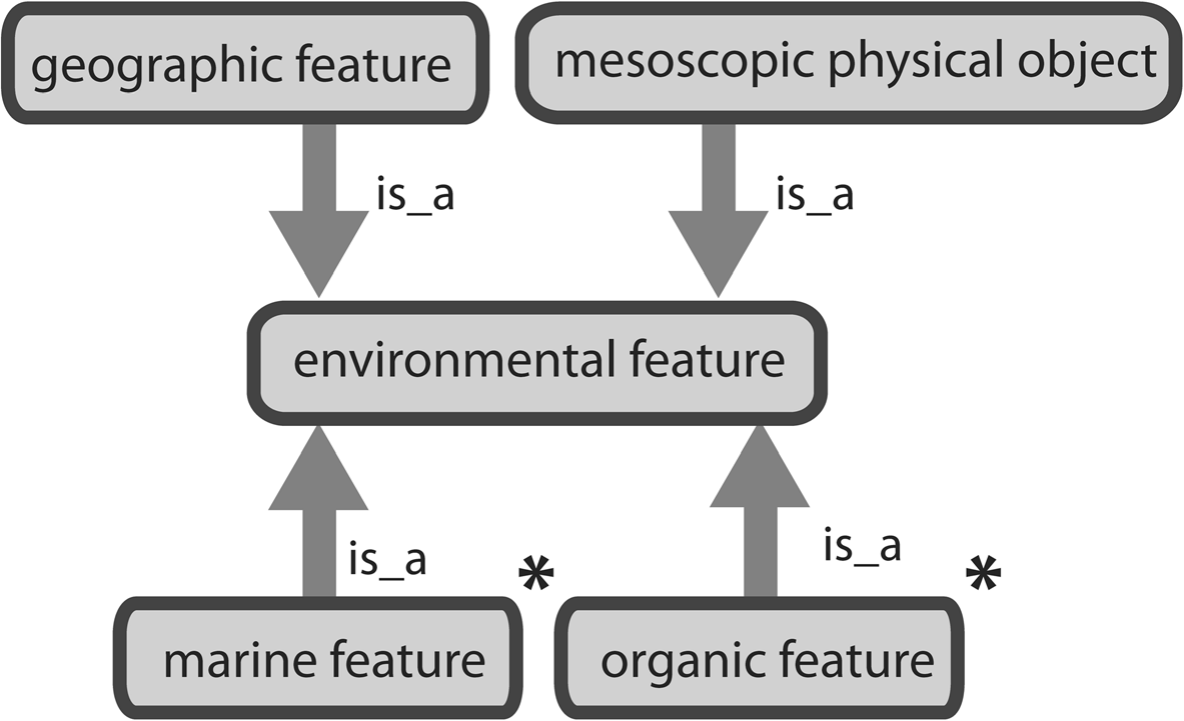
**ENVO’s feature hierarchy includes classes describing entities of geographic and mesoscopic scale.** Classes created during term capture exercises (*marine feature, organic feature*; marked with asterisks) temporarily house subclasses which will be curated and redistributed into more appropriate classes as needed.

ENVO’s *environmental feature* hierarchy comprises sub-branches addressing a number of spatial scales (Figure 2). Firstly, the *geographic feature* [ENVO_00000000] subclass contains subclasses that have been adapted from geographic surveys (e.g. those of the BGS and USGS). The current subclasses of *geographic feature* include *hydrographic feature* [ENVO_00000012], *physiographic feature* [ENVO_00000191], and *anthropogenic geographic feature* [ENVO_00000002] To promote interoperability with established geographic resources, many of ENVO’s *geographic feature* classes have synonyms which reference terms in geographic resources such as the USGS vocabularies, Alexandria Digital Library’s [32] Feature Type Thesaurus (FTT; [33]), the GeoNames geographical database’s [34] feature classes, and SWEET’s earthrealm ontologies [13]. The provenance of these synonyms is defined and cross-references to these terms will be added during curation of ENVO’s classes. Aside from geographic features, features that are of smaller spatial scale, such as carcasses and fomites, are included as subclasses of *mesoscopic physical object* [ENVO_00002004]. Lastly, two subclasses of *environmental feature*, *marine feature* [ENVO_01000031] and *organic feature* [ENVO_01000159], are also present to temporarily accommodate user requests. As described below, these will be curated and redistributed among the appropriate geographic or mesoscopic classes in due course.

ENVO’s *environmental material* hierarchy has less depth relative to those of *biome* and *environmental feature*. Broad subclasses such as *soil*, *water* [ENVO_00002006], and *sediment* [ENVO_00002007] are subdivided either by using well-known schemes (e.g. the United Nations Food and Agriculture Organization soil classification) or by referencing commonly used terms in the relevant domain following expert engagement.

Across ENVO’s hierarchies, lower-level branches grow primarily on the basis of requests from users and engagement with experts. The latter sometimes result in capture of large numbers of new classes from specific areas as branches expand quickly to accommodate community needs. Requests for new ontology classes are managed through the ENVO issue tracker [35]. After initial incorporation of new terms, branches may be restructured while textual and logical definitions are added or improved by curators.

### A brief annotation guide

The impact of ENVO will strongly depend upon the accurate use of the ontology during annotation, for example in the description of biological samples. Three of ENVO’s top-level classes – *biome*, *environmental feature*, and *environmental material* – allow for the non-redundant description of environments of a wide range of different sorts along three complementary dimensions. While it is possible to use a single class from any one of these hierarchies for annotation, a tripartite annotation will provide a more informative description. The examples below illustrate a recommended form for ENVO annotations.

As a first example, consider a killer whale (*Orcinus orca*) observed feeding near a subtidal rocky reef. One appropriate description would include three classes:from the *biome*, *environmental feature*, and *environmental material* hierarchies, respectively. Each class represents the surroundings of the entity of interest at a progressively more local scale, thereby offering complementary perspectives on the whale’s environment. While it may be argued that some classes are redundant (e.g. *coastal water* and *neritic epipelagic zone biome*), consider a killer whale swimming through *contaminated water* [ENVO_00002186], *brackish water* [ENVO_00002019], or *eutrophic water* [ENVO_00002224]. An explicit annotation of this sort offers the opportunity to compare observations of, e.g., whale ethology in different water types with fewer unexpressed assumptions and thus greater confidence.

*neritic epipelagic zone biome* [ENVO_01000042]

*marine subtidal rocky reef* [ENVO_01000150]

*coastal water* [ENVO_00002150]

To further illustrate the utility of multiple descriptors, consider the fruiting bodies of the Rogue mushroom (*Psathyrella aquatica*; [36]), which is the only mushroom species known to fruit underwater. Fruiting bodies were observed in the Rogue River (located in the Cascades ecoregion) in well-oxygenated and flowing river water, primarily on or near decaying wood (D. Southworth, R. Coffan, pers. comm., June 2010). A useful annotation for this case would include the ENVO classes *Small river biome* [ENVO_00000890] and *temperate coniferous forest biome* [ENVO_01000211]*;* the *environmental feature*, *river bed* [ENVO_00000384]; and the *environmental material* classes, *fresh water* [ENVO_00002011] and *wood* [ENVO_00002040]*.* This organism is an example of an entity appropriately described with multiple classes from ENVO’s hierarchies. If annotators are limited to one class from each hierarchy, they should select the class that captures that *biome*, *environmental feature*, or *environmental material* most causally relevant to the entity in question and that is the most specific available.

Currently, no formal relations between an entity of interest and the ENVO classes used to describe its environment are defined. These relations are necessary for semantically meaningful annotation and will be developed in the near future. Current considerations are described below. With respect to ENVO’s *biome* class, we will include a relation specializing BFO:*part of* [BFO_0000050] that is intended to indicate that the entity is strongly associated with a given biome class. For example, a conifer may stand in this relation to a *coniferous forest biome*. We shall also add a causally weaker relation derived from RO:*located in* [RO_0001025]. Continuing the example above, a day hiker may stand in this relation to a given *coniferous forest biome*. Relations between an entity of interest and subclasses of *environmental feature* are less straightforward; however, they are likely to reflect the degree to which the environment of an entity of interest is causally influenced by a given environmental feature. Finally, relations to *environmental material* will likely include sub-relations of RO:*surrounded by* [RO_0002219] such as “ventrally surrounded by” and “dorsally surrounded by” to capture, for example, the relations between a duck, water, and air. Some of these relations may come from the biological spatial ontology (BSPO; Dahdul et al., this issue). Relations pertaining to the *environmental condition* and *habitat* classes will be considered once these classes are better defined. Developments will be announced on the ENVO website [37].

### Adoption and use

ENVO has been adopted by or used in several projects. We describe a few examples below. A more complete list may be found on the ENVO website [38].

The omics community has been an early-adopter of ENVO, which is a recommended ontology in the core component of the Minimal Information about any (x) Sequence (MIxS) specification [39], a project of the Genomic Standards Consortium (GSC; [40]). MIxS-compliant sequence submissions to the International Nucleotide Sequence Database Collaboration (INSDC) will include one class from each of ENVO’s primary hierarchies. Retroactive annotation of genomic data has also been performed. For example, the Marine Ecological GenomiX portal (Megx.net; [41]) offers a manual annotation of a portion of the genome collection using classes from Habitat-Lite [42,43], a proper subset of ENVO designed for use in the genomic domain. The International Census of Marine Microbes (ICOMM) project offers more complete ENVO annotations for each of its constituent projects, using classes from the *biome*, *environmental feature*, and *environmental material* hierarchies. These annotations are searchable through the Visualization and Analysis of Microbial Populations Structures (VAMPS) environmental data search page [44]. Additionally, the Earth Microbiome Project (EMP; [45]) is currently employing ENVO classes to annotate thousands of samples from environmentally and biomedically motivated studies (See “EMP Sample Breakdown” [46]). Individual studies have also employed retroactive annotation to help evaluate the distribution of microbes using genomic data (e.g. [47]).

Outside the omics community, StrainInfo [48,49], a service which indexes and allows searching over numerous microbial culture collections, has used ENVO in its semantic representation of isolation environment [50]. Further, recent interaction with the Environments-EOL initiative [51], which is utilising text-mining approaches to annotate Encyclopedia of Life (EOL; [5]) pages with ENVO classes, is providing valuable guidance in ENVO’s development. Further, we have worked with the ecoinformatics community to map the environmental descriptors in ENVO to the SPIRE vocabulary [52]. This allows ecological interaction data mapped to SPIRE to be re-mapped to ENVO. Additionally, ENVO is being used as a standard vocabulary by the Encyclopaedia of Life (EOL) (C. Parr, pers. comm.).

As ENVO annotations become more widely available, databases and data retrieval tools are supporting queries over ENVO classes. For example, the Genomic Metadata for Infectious Agents Database (GEMINA; [53]) supports queries using ENVO classes, and the National Institute for Allergy and Infectious Diseases (NIAID) Bioinformatics Resource Centers (BRCs) use ENVO in formulating metadata pertaining to environmental material [54].

### Governance and consortium description

Due to its early adoption and use by the metagenomics community, ENVO has been accepted as a project within the framework of the Genomic Standards Consortium led by a small team of core developers [55]. The core team maintains the ontology while steadily aligning ENVO with the OBO Foundry principles [17,56]. This model will support ENVO’s use and development while promoting sustainable integration with other OBO ontologies such as the Gene Ontology (GO; [20]), the Phenotypic Quality Ontology (PATO), the multi-organism anatomy ontology (UBERON; [57]) and the Chemical Entities of Biological Interest (CHEBI; [58]) ontology. The wider ENVO consortium has developed primarily through workshops, meetings, and user engagement. The consortium includes a wide range of participants, including representatives from scientific domains such as biodiversity, biomedicine, microbiology, marine ecology, nutrition, long-term environmental research, and ethnogeography. Details of workshop attendance and contributions are currently hosted on the GSC wiki [59] and demonstrate the breadth of engagement in the project. Membership of the consortium is open and we welcome participation from any discipline with an interest in contextualising environmental data.

### Downloads

ENVO’s latest release version is available for download [60]. A file including only ENVO classes (envo-basic.obo) is available as well as files with additional classes from ontologies used to construct logical definitions in ENVO (envo.obo and envo.owl). The ontology is available both in OBO and OWL format. Currently, these formats are semantically equivalent; however, more expressivity may be added to the OWL format in future releases. The version of the ontology described in this manuscript is available from http://purl.obolibrary.org/obo/envo/releases/2013-09-24/envo.owl.

### Conclusions & outlook

ENVO is a community-led ontology that supports the representation of environments across and beyond the biological and biomedical domains. While work remains to be done in the definition of ENVO terms and relations as well as in gathering expert input across this large domain, we believe that ENVO offers an approachable and immediately useful resource to support researchers in the annotation of environmental features of their data.

In the near future, we aim to finalise the alignment of ENVO with BFO and add further classes such as ‘niche’. An additional goal is the creation of class-instance relations between environments and place names. This will be achieved by linking ENVO with GAZ, a first step towards an open source gazetteer constructed on ontological principles [61]. When linked with ENVO descriptors, GAZ will provide a basis to infer environment from place names and, through this, from other geospatially annotated data. Lastly, continuing outreach activities will focus on supporting initiatives that have expressed an interest in using ENVO (for example EnvDB [62]) as well as engaging new users and contributors.

On behalf of the consortium, we invite those interested in contributing to, co-developing, or using ENVO to contact us through the project website [63]. In particular, we welcome the input of expert ecologists in the definition and resolution of classes such as biome, habitat, and niche and of expert geographers who can help us with the integration of additional terms commonly used when describing environments. Furthermore, we invite domain experts, working with specific environment-types, to contribute their knowledge in the development of the relevant branches of the ontology.

## Methods

ENVO is developed using the OBO-Edit ontology development tool [64]. This tool allows the creation and maintenance of ontologies in OBO-Format [65], which is an alternative syntax for a subset of the Web Ontology Language (OWL).

The ENVO editorial team consults a variety of sources when creating and editing terms, including the ENVO request tracker. The core ontology is maintained in OBO-Format in a subversion repository hosted on Google Code [22]. Each change to the ontology triggers a centralized ontology-based Continuous Integration server (Mungall et al., unpublished) to perform a series of checks^c^. These include lexical checks (for example, ensuring that no two classes have the same unique label) as well as logical checks, executed using the Elk reasoner [66]. We use the Elk reasoner because it is fast, and the current version of ENVO does not currently make use of any OWL constructs that fall outside of the EL++ subset of the OWL language. We use the OBO Ontology Release Tool (OORT; [67]) as a general framework for performing OBO-Format to OWL conversion and execution of reasoner checks.

We also use OORT for building public releases of ENVO. Each public release consists of both OBO Format and OWL versions of the ontology, as well as a number of subsets, including the ENVO-lite subset. Note that currently the OBO and OWL versions of the ontology are semantically identical, but in future we may make use of a wider range of OWL constructs, in which case the OBO version will be a subset of the OWL version. The main public release of ENVO incorporates a subset of classes from external ontologies (CHEBI, PATO) – we also make available a “basic” subset that excludes external ontologies and references to them. For each release, the ontology is pre-classified automatically, using Elk running within the OORT environment. This allows us to leverage external ontologies such as CHEBI.

The current version of the ontology makes use of 127 EquivalentClasses axioms (for example, ENVO_0002119 ‘alkaline hot spring’ has an equivalence axiom to an OWL construct that is the class intersection of ‘hot spring’ (ENVO_0000051) and the existential restriction has_quality some ‘alkaline’ (PATO_0001430). Currently we only have a handful of disjointness axioms in the ontology – we are experimenting with making pairs of classes disjoint and ultimately moving toward jointly-exhaustive pairwise-disjoint class hierarchies.

## Endnotes

^a^Note that we write the URLs identifying ontology classes in an abbreviated form – to obtain the full URL, add the prefix: http://purl.obolibrary.org/obo/

^b^BFO itself is currently undergoing revision (the draft specification of BFO 2.0 is available at http://bfo.googlecode.com/svn/trunk/docs/bfo2-reference/BFO2-Reference.docx), thus this alignment is work-in-progress.

^c^The system is available at http://build.berkeleybop.org/job/build-envo/

## Abbreviations

BGS: British Geographic Survey; BSPO: Biological spatial ontology; CHEBI: Chemical entities of biological interest; ENVO: Environment ontology; EOL: Encyclopedia of life; FTT: Feature type thesaurus; GEMINA: Genomic Metadata for Infectious Agents Database; GCMD: Global change master directory; ICOMM: The International Census of Marine Microbes; INSDC: International Nucleotide Sequence Database Collaboration; MIxS: Minimal information about any (x) sequence; OBI: Ontology for biomedical collections; OBO: Open biological and biomedical ontologies; OORT: OBO ontology release tool; OWL: Web ontology language; PATO: Phenotypic quality ontology; PCO: Population and community ontology; SWEET: Semantic Web for Earth and Environmental Terminology; SERONTO: Socio-Ecological Research and Observation Ontology; USGS: United States Geographic Survey; VAMPS: Visualization and analysis of microbial populations structures.

## Competing interests

The authors declare that they have no competing interests.

## Authors’ contributions

PLB co-develops and co-leads the ENVO project, with particular focus on the ontology’s marine branches, and wrote the manuscript. NM initiated and co-leads the ENVO project, has organised and chaired all of the ENVO workshops, has been the lead editor on early revisions of the ontology, has coordinated outreach of the project collecting user requirements and interacting with key project stakeholders (e.g. GSC, StrainInfo, ENA, EoL). BS was involved in initial creation of ENVO and leads the alignment of ENVO with BFO. CJM co-develops the ontology, is the acting release manager, and coordinates interaction with other ontologies. SEL assisted in the organization of the workshops and co-develops the ontology with focus on cross-links with PATO. All authors read and approved the final manuscript.
